# Anomalous mechanical materials squeezing three-dimensional volume compressibility into one dimension

**DOI:** 10.1038/s41467-020-19219-5

**Published:** 2020-11-05

**Authors:** Xingxing Jiang, Maxim S. Molokeev, Liyuan Dong, Zhichao Dong, Naizheng Wang, Lei Kang, Xiaodong Li, Yanchun Li, Chuan Tian, Shiliu Peng, Wei Li, Zheshuai Lin

**Affiliations:** 1 Technical Institute of Physics and Chemistry, Chinese Academy of Sciences, Beijing, 100190 China; 2 Center of Materials Science and Optoelectronics Engineering, University of Chinese Academy of Sciences, Beijing, 100049 P.R. China; 3 Laboratory of Crystal Physics, Kirensky Institute of Physics, SB RAS, Krasnoyarsk, 660036 Russia; 4 Department of Physics, Far Eastern State Transport University, Khabarovsk, 680021 Russia; 5 Siberian Federal University, Krasnoyarsk, 660041 Russia; 6 Wuhan National Laboratory for Optoelectronics and School of Physics, Huazhong University of Science and Technology, Wuhan, 430074 China; 7 Laboratory of Space Astronomy and Technology, National Astronomical Observatories, Chinese Academy of Sciences, Beijing, 100101 China; 8 University of Chinese Academy of Sciences, Beijing, 100049 China; 9 Beijing Synchrotron Radiation Facility, Institute of High Energy Physics, Chinese Academy of Sciences, Beijing, 100049 China; 10 Institute of Deep-sea Science and Engineering, Chinese Academy of Sciences, Sanya, 572000 China; 11 Institute of Mechanics, Chinese Academy of Sciences, Beijing, 100190 China; 12 School of Materials Science and Engineering; TKL of Metal and Molecule-Based Material Chemistry, Nankai University, Tianjin, 300350 China

**Keywords:** Mechanical properties, Mechanical properties

## Abstract

Anomalous mechanical materials, with counterintuitive stress-strain responding behaviors, have emerged as novel type of functional materials with highly enhanced performances. Here we demonstrate that the materials with coexisting negative, zero and positive linear compressibilities can squeeze three-dimensional volume compressibility into one dimension, and provide a general and effective way to precisely stabilize the transmission processes under high pressure. We propose a “corrugated-graphite-like” structural model and discover lithium metaborate (LiBO_2_) to be the first material with such a mechanical behavior. The capability to keep the flux density stability under pressure in LiBO_2_ is at least two orders higher than that in conventional materials. Our study opens a way to the design and search of ultrastable transmission materials under extreme conditions.

## Abstract (web summary)

Anomalous mechanical behaviors provide an opportunity to regulate the functions of materials. Here the authors show that materials with coexisting negative, zero and positive compressibilities can “squeeze” volume compressibility into one direction, and thus stabilize transmission processes under pressure.

## Introduction

The anomalous mechanical materials provide a novel approach to modulate the functional properties that is inaccessible by conventional materials, owing to their counterintuitive squeeze-expanded^1^, stretch-densified^2^, push/pull-twisted^3^, or negative-modulus^4^ response. In particular, the control of compressibilities in these materials can precisely regulate the physical properties under high-pressure conditions, which shows wide application prospects in, e.g., superconductivity modulation^5^, ferroelectricity enhancement^6^, ultrasensitive sensing^7^, ultrastable information communication^2,8^, energy harvesting^9^, seismic wave detection^1,10^, and body armor fabrication^11,12^. As many physical properties are tightly related to the transmission processes of electrons, photons, and phonons^13^, the transmission stability under pressure is essential to improve the performances of functional materials in extreme and complex environments, but the relevant studies in anomalous mechanical materials have been elusive.

In a transmission process, the energy or particle flux density is determined by the transmission power and the cross section vertical to the propagating direction in the material^14^. In order to stabilize the transmission processes under pressure, the cross section, normally contracted with the increase of ambient pressure, needs to be kept as constant as possible to resist the influence of pressure fluctuation Δ*P*. To this end, a most effective approach is to make the linear compressibility (*α*_*l*_ = −(1*/l*)(Δ*l/*Δ*P*)) along the propagating direction ${\hat{\boldsymbol{l}}}$ exactly equal to the volume compressibility (*α*_*V*_ = −(1*/V*)(Δ*V/*Δ*P*), V is volume), i.e., to squeeze the three-dimensional volume compressibility into one dimension. Under this condition, the area compressibility on the cross section perpendicular to the ${\hat{\boldsymbol{l}}}$ direction is kept zero under pressure so as to remove the influence of Δ*P*, considering that the volume compressibility is identically equal to the sum of linear compressibilities for any three orthogonal axes^15^. Note that for a specific material volume compressibility is a constant which can be represented by a scalar without directivity, while linear compressibility is a “vector” depending on direction. The “vectorization” would endow volume compressibility with the “directionality” to subtly manipulate physical properties. However, this mechanical matching condition is impossible to be realized in conventional materials which are shrinked along arbitrary axis under pressure (i.e., exhibiting positive linear compressibility, or PLC, behavior in arbitrary direction), since the volume compressibility is always larger than the linear compressibility along any direction in these materials. This problem can be solved in anomalous mechanical materials with negative linear compressibility (NLC) and/or zero linear compressibility (ZLC).

NLC and ZLC materials possess the pressure-responding behavior that the size along a specific direction expands^2,16^ and keeps constant^17^, respectively, when being squeezed by hydrostatic pressure. These counterintuitive pressure-responding behaviors violate the commonly well-recognized “compression-contraction” effect, and have promising applications in many advanced technologies, e.g., in ultra-sensitive pressure sensors^2^, deep-sea optical communications^8^ and smart body armors^12^. After some mathematical deductions (see Sect. S1), we conclude that: (i) when an NLC axis is introduced into the normal mechanical system, the condition for squeezing three-dimensional volume compressibility into one dimension, i.e., the exact matching between volume and linear compressibilities, can be achieved only if the absolute value of NLC coefficient is larger than either of the PLC coefficients. Nevertheless, this matching condition is seldom satisfied in practice, because in almost all known NLC materials the NLC coefficient is smaller than the PLC values. (ii) when a ZLC axis is introduced, the matching condition cannot be satisfied as well. (iii) when PLC, NLC, and ZLC are independently coexisted in the system, the matching direction can be always found.

However, both NLC and ZLC were separately discovered only in a handful of materials^2,17^ and the coexistence of NLC, ZLC, and PLC has never been demonstrated in any material. From the viewpoint of molecular geometry, the NLC behavior frequently occurs in the one-dimensional chains or two-dimensional layers with wrinkles, which can be flattened by the extrusion of neighboring counterparts to generate the squeeze-expanded behavior under pressure, as summarized in the famous Lifshitz mechanism^18,19^. On the other hand, the ZLC behavior has been observed in the graphite structure with the flat and strongly covalent two-dimensional layers constructed by the three-coordinated carbon atoms, which almost keeps constant up to the phase-transition pressure with the linear compressibility along the a(b)-axis almost equals to that of diamond^20^. Inspiring from these studies, we speculate that, if the graphite layers become corrugated along a specific axis, the wrinkles in layers would be flattened by the extrusion of neighbor layers, and thus the NLC can be induced along this direction. The corrugated-graphite-like (CGL) architecture at a molecular level, therefore, would achieve the coexistence of NLC, ZLC, and PLC in a single material.

Analogous to carbon atoms in graphite, boron atoms can be three-coordinated with oxygen to form the strongly covalent [BO_3_] groups. The planar [BO_3_] groups are likely to further connect with other groups to form infinite two-dimensional layers^21,22^, by which the anomalous mechanical properties have been observed, e.g., in KBe_2_BO_3_F_2_^23^ and in AEB_2_O_4_^17^.

In this work, by applying the CGL model to borates, we identify that lithium metaborate (LiBO_2_) possesses the simultaneous PLC, ZLC, and NLC behaviors from 0 to 2.52 GPa, so the volume compressibility can be squeezed into one dimension. The capability of flux density stability in LiBO_2_ is higher by at least two orders than conventional materials. Meanwhile, LiBO_2_ exhibits a good optical transparency from deep-ultraviolet (DUV) to near-infrared (IR) region, manifesting its promising prospect for ultrastable optical transmission in high-pressure environments.

## Results

### Crystal structure

**Fig. 1:**
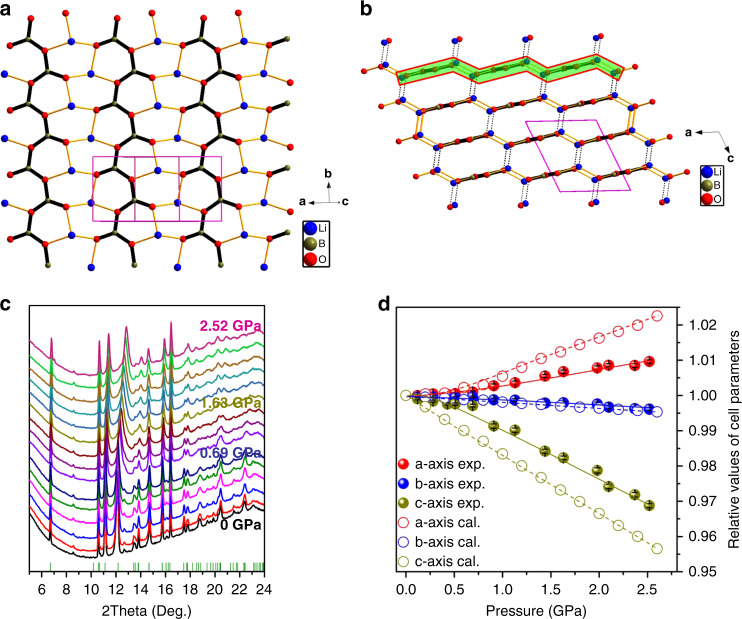
Crystal structure and linear compressive behaviors in LiBO_2_. **a** Graphite-like [LiBO_2_]_∞_ layer (in the a-b plane), in which the [BO_2_]_∞_ belt is highlighted by thick bars in a zig-zag configuration. **b** Bond connection between the neighbor [LiBO_2_]_∞_ layers (along the c-axis), in which the corrugations introduced by (LiO_3_) bases are highlighted by green strips. The unit cells are represented by pink boxes. **c** evolution of the high-pressure XRD patterns. **d** Variations of *a*-axis, *b*-axis, and *c*-axis with respect to hydrostatics pressure. Experimental and first-principles results are represented by solid and open circles, respectively.

Our refined structure analysis reveals that LiBO_2_ crystalizes in monoclinic P2_1_/c space group at ambient pressure, in which boron and lithium atoms are threefold and fourfold coordinated with oxygen atoms to form [BO_3_] triangles and [LiO_4_] triangular pyramids, respectively, as firstly determined by Zachariasen^24^. By sharing the corner oxygen atoms, the [BO_3_] triangles are connected with each other, giving rise to an infinite [BO_2_]_∞_ belt along the b-axis (Fig. 1a). The [BO_2_]_∞_ belts are bounded by the (LiO_3_) bases in [LiO_4_] triangular pyramids along the a-axis to construct the [LiBO_2_]_∞_ layer, in which the hexatomic [Li_2_BO_3_] and [LiB_2_O_3_] rings are alternatively connected to form a graphite-like-layered architecture. The [LiBO_2_]_∞_ layers are further adhered to one another along the c-axis by the Li–O bonds out of the (LiO_3_) bases (Fig. 1b). Note that the introduction of nonplanar (LiO_3_) bases produces wrinkles along the a-axis in the [LiBO_2_]_∞_ layer, and thus forming a typical CGL structure.

### Coexistence of NLC, ZLC, and PLC

The high-pressure XRD demonstrates that the single phase of LiBO_2_ is well preserved from 0 to 2.52 GPa, as no new XRD peak emerges or vanishes (Fig. 1c and Fig. S2). The extracted cell parameters (Fig. 1d) reveal that the crystallographic a-axis is anomalously elongated by about 1% in the measured pressure range, indicating the NLC behavior along this direction, while the b-axis remains nearly constant and only contracts by 0.4%. In comparison, the c-axis experiences very prominent shrinkage of 3.1%. The corresponding linear compressibilities along the mechanical principal *X*-, *Y*- and *Z*- axes are −5.33(34)/TPa, 1.66(11)/TPa, and 25.63(97)/TPa, respectively (The transformation matrix between crystallographic and mechanical principal axes see Table S3). Note that the compressibility along the Y-axis is much smaller than that in the majority of materials and can be categorized into ZLC^25,26^. Therefore, the NLC, ZLC, and PLC are simultaneously presented in LiBO_2_. In principle, one may always find the ZLC direction in an NLC material by determining the specific direction between NLC and PLC axes. However, the existence of NLC, PLC, and ZLC axes in LiBO_2_ is entirely independent of each other.

### Mechanism elucidation

**Fig. 2:**
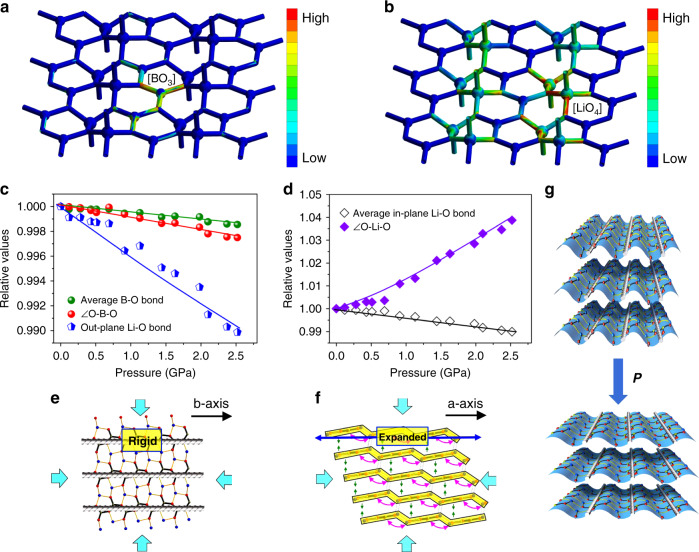
Microscopic mechanism for the simultaneous NLC, ZLC, and PLC in LiBO_2_. **a** Stress and **b** Strain distribution in LiBO_2_ structure, respectively, by finite element analysis. **c**, **d** Pressure-induced modification of bond lengths and bond angles responsible for ZLC and NLC. **e** Schematic for the ZLC behavior. **f** Schematic for the NLC behavior. **g** Schematic for the mechanism of the pressure-responding behaviors originated from the CGL structure. The [BO_2_]_∞_ chains are represented by gray bars and the [LiO_3_] bases are represented by blue corrugations.

To shed light on the microscopic mechanical mechanism in LiBO_2_, the computational simulations were performed. The finite element analysis (FEA) reveals that most of the stress under pressure is undertaken by [BO_3_] triangles (Fig. 2a), indicating that the quasi-rigid [BO_2_]_∞_ belts play the key role to resist the external pressure. In comparison, the strain is concentrated on [LiO_4_] triangular pyramids (Fig. 2b), demonstrating that the deformation in these microscopic units dominantly determine the pressure-induced structural modification in LiBO_2_.

Because the light lithium and boron atoms have low X-ray scattering factor, it is very difficult to accurately determine their atomic position variation by high-pressure XRD. In order to overcome this problem, the first-principles simulations with high precision were adopted. The calculated compressibilities along the *X*-, *Y*- and *Z*-axes are −14.50(51)/TPa, 1.77(01)/TPa, and 38.47(70)/TPa, respectively (Fig. 1d and Table S2), in well consistence with the experimental values. The atomic simulations (see Table S4) reveal that, from 0 to 2.52 GPa, the average B–O bond length and ∠O-B-O angle in the [LiBO_2_]_∞_ layer only decrease by 0.1% and 0.2%, respectively (Fig. 2c, e), confirming the large rigidity of the [BO_2_]_∞_ chains (along the *Y*- or the *b*-axis) formed by [BO_3_] triangles. This makes LiBO_2_ exhibit a ZLC behavior along this direction, similar to the role of carbon triangles to ZLC in graphite. Since the covalent strength of B–O bond is smaller than that of C–C bond, the ZLC effect in LiBO_2_ is weaker than that in graphite (~0.7/TPa)^20^ and diamond (0.75/TPa)^27^. Owing to the rigidity of [BO_2_]_∞_ chains, the mechanical strain principally occurs at the [LiO_4_] triangular pyramids, and thus results in different mechanical behaviors along the *X*- (nearly the *a*-) and *Z*-axes: On one hand, the Li–O bonds protruding outside the [LiBO_2_]_∞_ layers are significantly contracted by ~1.0% (Fig. 2c), which gives rise to the normal PLC along the *Z*-axis. On the other hand, the vertical stress from the approaching of neighbor [LiBO_2_]_∞_ layers makes the [LiO_3_] bases (i.e., the wrinkles in the CGL architecture) flatten with the ∠O-Li-O angles dramatically opened by 3.8% (Fig. 2d). This results in the expansion of [LiBO_2_]_∞_ layers in response to the increase of hydrostatic pressure, which transcends the contraction of intralayer Li–O bonds (~1.0%) caused by the in-plane strain, and eventually give rise to the net NLC behavior along the *X*-axis (Fig. 2f). Therefore, the corrugated [LiBO_2_]_∞_ layers with CGL architecture is the structural origin for the simultaneous occurrence of NLC, ZLC, and PLC in LiBO_2_, as depicted in Fig. 2g.

### Application prospects from mechanical properties

**Fig. 3:**
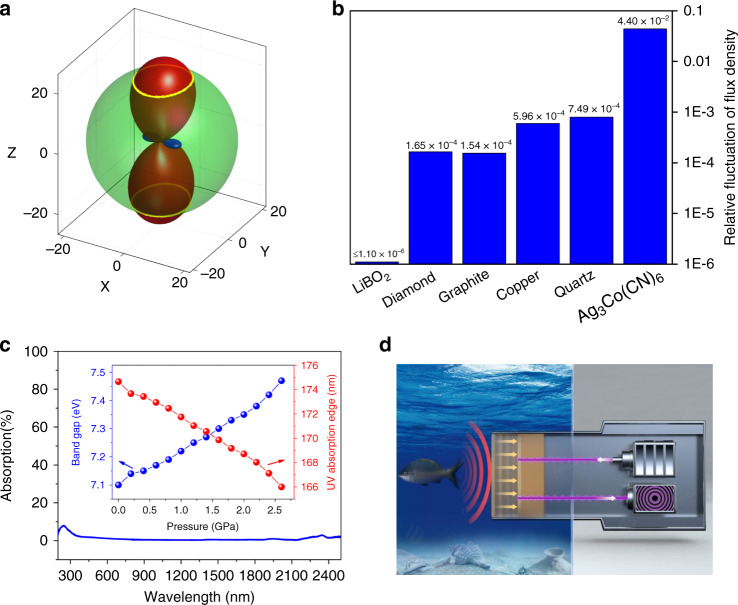
Flux density stability, optical property, and potential application of LiBO_2_. **a** The matching between linear and volume compressibilities. Positive and negative linear compressibilities are represented by red and blue surfaces, and volume compressibility is represented by green sphere, respectively. The isoline between linear and volume compressibilities is highlighted by yellow curve. **b** Comparison of the relative fluctuations of flux density among LiBO_2_ and other materials, as they are moved from the sea level to Mariana Trench. **c** UV–Vis diffuse-reflectance spectrum. The calculated dependence of pressure vs. UV absorption edge (i.e., band gap) is shown in the inset. **d** Schematic for the ultrastable optical detection apparatus fabricated by LiBO_2_ in the deep-sea environment.

Figure 3a plots the exact curve squeezing three-dimensional volume compressibility into one dimension in LiBO_2_. In comparison, owing to the large discrepancy between volume and linear compressibilities the matching curves do not exist in Ag_3_Co(CN)_6_ (the material with largest NLC)^8^, diamond^27^ and graphite^20^ (the ZLC materials), and the most used transmission materials, e.g., copper^28^ and quartz^29^ (Fig. S5). This indicates that LiBO_2_ has the superior capability to alleviate the influence of pressure on the flux density stability compared with other materials. Taking the deep-sea environment as an example, when moved from the sea level to the Mariana Trench (the deepest trench on earth with the depth of ~11,000 m), the relative fluctuations of flux density in Ag_3_Co(CN)_6_, diamond, graphite, copper, and quartz are at least two orders larger than that in LiBO_2_ (Fig. 3b), on the condition that the resolution for the compressibility-angle-tuning is one degree (see the detailed discussion in Tables S5 and S6). Therefore, LiBO_2_ can bring out great advantage in the ultrastable transmission apparatus in high pressure-fluctuating environments. Moreover, the UV–Vis diffuse-reflectance spectrum reveals that LiBO_2_ has very good optical transparency in the range from DUV to near-IR (Fig. 3c). The further first-principles calculations show that the UV absorption edge of LiBO_2_ (175 nm under atmospheric pressure) is blue-shifted as pressure increases (inset in Fig. 3c and Table S7), demonstrating that the wide optical transmittance in LiBO_2_ is well kept under pressure. Therefore, from a proof-of-principle perspective LiBO_2_ exhibits a promising prospect to be used in the ultra-precise optical detection apparatus in high pressure-fluctuating environments, e.g., in deep-sea, as schematically depicted in Fig. 3d. Independent of the deep-sea pressure, the optical cross-section area in LiBO_2_ can almost keep constant, and thus the fluctuation of optical flux density owing to the ambient pressure is eliminated and the signal from detection target is transmitted with ultra-high fidelity. Doubtlessly, to realize these practical applications, there is still a long way to go for overcoming lots of technical hurdles, such as large-size single crystal growth and water-proof treatment, which, however, we believe can be eventually solved with the development of advanced techniques.

## Discussion

Aiming at stabilizing flux density under pressure, we have exhibited a rational materials design from physical and mathematical derivation, structural model proposition to material discovery, and LiBO_2_ was discovered as the first material, to the best of our knowledge, with coexisting NLC, ZLC, and PLC to “vectorize” volume compressibility into the linear compressibility in a specific direction. By virtue of this fascinating mechanical property in combination with excellent optical transparency, LiBO_2_ holds great promise in the ultrastable optical apparatus under high pressure-fluctuating conditions. More importantly, both pressure and volume are involved in the equation of state and determine the basic thermodynamic functions of state (e.g., enthalpy and free energy). The “vectorization” of volume compressibility, the physical quantity directly connecting these two-state parameters, would greatly improve the tuning ability of pressure on the functional properties and may provide a freedom to modulate the performance of materials related to the transmission of electrons, photons and phonons, such as electric conductivity, optical waveguide, and thermal conductivity.

## Methods

### Sample preparation

Polycrystalline LiBO_2_ was synthesized through solid-state reaction in stoichiometric ratio with analytically pure Li_2_CO_3_ (99.99%) and B_2_O_3_ (99.98%) purchased from Sigma-Aldrich corporation as the starting materials. These ingredients were mixed homogeneously, then heated with the heating rate 30 °C/h in a muffle furnace. The ingredients were taken out and carefully grinded at 200 °C, 400 °C, and 600 °C. Finally, the temperature was raised to 700 °C and kept for 24 h. After cooling to room temperature, the white powder of target compound was obtained.

### High-pressure X-ray diffraction

The high-pressure XRD experiment was conducted in the 4W2 beam line with the radiation at 0.6199 Å of Beijing Synchrotron Radiation Facility (BSRF). The X-ray beam at the wavelength 0.6199 Å was focused into a 36 × 12 μm^2^ spot using Kirkpatrick–Baez mirrors. The hydrostatic pressure was exerted by the systematic diamond anvil cells (DAC) with the culet diameter of 400 μm. The sample in well grinded powder was placed in a hole of about 120 μm diameter in a pre-indented stainless steel gasket with the thickness of 40 μm. Silicon oil was adopted to act as the pressure-transmitting medium and ruby chips were placed for pressure calibration by measuring the fluorescence shift as a function of pressure^30^. The diffraction patterns were recorded by a Pilatus image plate and integrated with the FIT2D software package. The pressure-depending lattice parameters were refined by Rietveld refinement^31^ using TOPAS software^32^. The compressibility were fitted by the PASCal software^33^.

The determination of cell parameters under high pressure is influenced by the XRD peak-broadening due to the pressure inhomogeneity from pressure-transmitting medium. It is well known that the hydrostatic-pressure limit of silicon oil is about 0.9 GPa^34^, which is lower than the upper-pressure limit of 2.52 GPa in this work. At the first glance, the use of silicon oil would deteriorate the accuracy of the refined cell parameters. However, as displayed in Table S1, the broadness of peaks just affects the third decimal place (i.e., in the magnitude of 0.003 Å), although the error of the cell parameters is increased by about three times (taking the *a*-axis as an example, from 0.0009 to 0.0027 Å) when pressure increases from 0 to 2.52 GPa. In comparison, the pressure-induced change of the cell parameters is in the magnitude of 0.02 Å, one-order higher than the influence of the error. Therefore, the peak-widening due to the pressure inhomogeneity from silicon oil does not affect the conclusion that NLC, ZLC, and PLC behaviors are coexisted in LiBO_2_.

### Raman spectrum

The Raman pattern was recorded from 100 to 2000 cm^−1^ at room temperature, using in Via-Reflex, equipped with a solid-state laser with a wavelength of 532 nm. In order to improve the signal to noise ratio of the spectrum, 10 integrations were carried out with an integration time of 10 s at a resolution of 1 cm^−1^.

### Infrared spectrum

The infrared spectrum was obtained at room temperature in a range from 400 to 2000 cm^−1^ with resolution of 1 cm^−1^ via a Bio-Rad FTS-60 FTIR spectrometer. The sample and dried KBr (5 mg of the sample, 500 mg of KBr) were mixed thoroughly together.

### UV–Vis Diffuse Reflectance Spectroscopy

The diffuse reflectance data were collected with a Varian Cary 7000 UV–vis−NIR spectrometer equipped with an integrating sphere in the wavelength range from 200 to 2500 nm. BaSO_4_ was employed as the 100% reflectance standard.

### Finite element analysis (FEA)

FEA was performed by ANSYS Workbench software. The [BO_3_] and [LiO_4_] groups were respectively modeled by four and five rigid balls (modeling the atoms) connected by three and four small bars (modeling the bonds) with the same diameter. Based on the spacial arrangement of the microscopic groups in the crystal lattice, the whole architecture was built by 36 [BO_3_] and 36 [LiO_4_] units as depicted in Fig. S1. Since the characteristic vibrational frequencies of B–O bond in [BO_3_] and Li–O in [LiO_4_] groups are about 1500 cm^−1^ and 400 cm^−1^ (refs. ^35,36^), the elastic coefficient ratio between the two types of bonds are about 22:1, owing to the formula $k = m{\upomega}^2$, where *k*, ω, and *m* are elastic coefficient, vibrational frequency, and atomic mass, respectively. The elastic modulus of the balls (modeling the atoms) was set to 10^6^ orders higher than that of B–O bonds in order to manifest their rigidity. The FEA model was meshed into 80758 tetrahdral elements which are connected by 161,243 nodes, and the hydrostatic pressures of 2.52 GPa was applied on the surfaces of the model. Finally, by solving the Cauchy continuum mechanics equations, the von-Mises equivalent stress and strain can be retrieved. As periodic boundary condition in the crystal lattice was not considered in the FEA simulation, to eliminate the boundary effect, the stress and strain distributions near center part were focused on to investigate the mechanical mechanism (Fig. S1).

### First-principles calculations

First-principles calculation was performed by CASTEP^37^, a plane-wave pseudopotential total energy package based on density functional theory (DFT)^38,39^. The functionals developed by Perdew, Burke, and Ernzerhof (PBE)^40^ in generalized gradient approximation (GGA)^41^ form were adopted to model the exchange-correlation terms in Hamiltonian. The optimized ultrasoft pseudopential^42^ were used to model the effective interaction between the valence electrons and atom cores, which allow us to use a small plane basis set without compromising the accuracy required by the calculation. High kinetic energy cutoff of 300 eV and Monkhorst-pack^43^ *k*-point mesh spanning <0.03 Å^−1^ in the Brillouin zone were chosen.

The theoretical cell parameters at various pressures were calculated by geometry optimization with both cell parameters and atomic position relaxed under different hydrostatic pressures, in which the Broyden–Fletcher–Goldfarb–Shanno^44^ minimization scheme was used. The convergence criteria for energy, maximum force, maximum stress and maximum displacement were set as 10^−5^ eV/atom, 0.03 eV/angstrom, 0.05 GPa, and 0.001 Å, respectively. The compressibilities based on the theoretical cell parameters were also fitted by the PASCal software^33^. The constituent atoms in LiBO_2_ are very light and their atomic scattering factor is very low, especially for lithium. The structural modification induced by pressure cannot be accurately determined by experiments only (see the experimentally refined CIF files in SI). The variation of atomic positions was determined by first-principles geometry optimization with the cell parameters fixed at the experimental values. For the electronic band structure calculation of LiBO_2_ the hybridized PBE0 functionals^45^ were adopted. Our previous studies have demonstrated that this type of functionals can accurately predict the band gaps of UV and deep-UV borates^46,47^. Raman and infared spectra were calculated by linear response method^48^. In band gap prediction and Raman/infrared spectrum calculation, norm-conserving pseudopotential^49^ and kinetic energy cutoff of 900 eV were used.

## Supplementary information

Supplementary Information

Description of Additional Supplementary Files

Supplementary Data 1

## Data Availability

The authors declare that all the data supporting the findings of this study are available from the authors
