# Supplementary material for: Anomalous mechanical materials squeezing three-dimensional volume compressibility into one dimension
Source: Nat Commun. 2020 Nov 5;11:5593. doi: 10.1038/s41467-020-19219-5 (PMC7644688; doi:10.1038/s41467-020-19219-5)
Supplement: Supplementary file 1 — Supplementary Information [file 41467_2020_19219_MOESM1_ESM.pdf]

Supplementary Information for

Anomalous Mechanical Materials Squeezing Three-Dimensional Volume Compressibility into One Dimension

Xingxing Jiang[1,2], Maxim S. Molokeev[3,4,5], Liyuan Dong[6], Zhichao Dong[7], Naizheng Wang[1,8], Lei Kang [1,2], Xiaodong Li[9], Yanchun Li[9], Chuan Tian[10], Shiliu Peng[11], Wei Li[12], Zheshuai Lin[1,2,8]*

[1]Technical Institute of Physics and Chemistry, Chinese Academy of Sciences, Beijing 100190, China, Email: zslin@mail.ipc.ac.cn.
[2]Center of Materials Science and Optoelectronics Engineering, University of Chinese Academy of Sciences, Beijing100049, P.R. China.
[3]Laboratory of Crystal Physics, Kirensky Institute of Physics, SB RAS, Krasnoyarsk 660036, Russia.
[4]Department of Physics, Far Eastern State Transport University, Khabarovsk 680021, Russia.
[5]Siberian Federal University, Krasnoyarsk 660041, Russia.
[6]Wuhan National Laboratory for Optoelectronics and School of Physics, Huazhong University of Science and Technology, Wuhan 430074, China.
[7]Laboratory of Space Astronomy and Technology, National Astronomical Observatories, Chinese Academy of Sciences, Beijing 100101, China
[8]University of Chinese Academy of Sciences, Beijing 100049, China.
[9]Beijing Synchrotron Radiation Facility, Institute of High Energy Physics, Chinese Academy of Sciences, Beijing 100049, China.
[10]Institute of Deep-sea Science and Engineering, Chinese Academy of Sciences, Sanya 572000, China.
[11]Institute of Mechanics, Chinese Academy of Sciences, Beijing 100190, China.
[12]School of Materials Science and Engineering; TKL of Metal and Molecule-Based Material Chemistry, Nankai University, Tianjin 300350, China.

Section S1 Matching condition between linear compressibility and volume compressibility

If there exists a matching direction (θ, φ) along which the linear and volume compressibilities coincide in a material, then the following condition must be satisfied:

$$\alpha(\theta,\varphi) = \alpha_V = \alpha_X+\alpha_Y+\alpha_Z \tag{1}$$

where $\alpha(\theta,\varphi)$ is determined by the spatial ellipsoid with the principal axes of α_X, α_Y, and α_Z, and varies in the range [minimum($\alpha_X,\alpha_Y,\alpha_Z$), maximum($\alpha_X,\alpha_Y,\alpha_Z$)]. Clearly, for a normal mechanical system where all three principal axes are PLC, $\alpha(\theta,\varphi)$ is always smaller than $\alpha_X+\alpha_Y+\alpha_Z$. The equation (1) cannot be fulfilled and the matching direction cannot be found.

Moreover, the matching conditions for the anomalous linear compressibilities are shown as follows:

(i) if only a NLC axis is introduced into the normal mechanical system, say, $\alpha_X < 0$, then the range of $\alpha(\theta,\varphi)$ value now is changed to

$$\alpha(\theta,\varphi) \le \text{maximum}(\alpha_Y,\alpha_Z) \tag{2}$$

If the equation (1) is satisfied, then $\alpha_X+\alpha_Y+\alpha_Z \le$ maximum(α_Y,α_Z), namely, $\alpha_X \le$ -minimum(α_Y,α_Z). Therefore, in the NLC system only that $|\alpha_X| \ge$ minimum(α_Y,α_Z), the direction (θ, φ) for $\alpha(\theta,\varphi) = \alpha_V$ can be found. However, the absolute value of the NLC component is smaller than that of either PLC component in majority of NLC materials, and thus this matching condition is seldom satisfied in practice. Although this matching condition could be achieved if NLC occurs in two-dimension, its

practical application would be hindered by the weak angle tenability owing to the low anisotropy within the two-dimensional negative compressibility plane.

(ii) if only a ZLC axis is introduced into the normal mechanical system, say, $\alpha_X = 0$, then

$$\alpha(\theta,\varphi) \leq \text{maximum}(\alpha_Y,\alpha_Z) < \alpha_Y+\alpha_Z \qquad (3)$$

Equation (1) cannot be fulfilled and the matching direction cannot exist. For the zero area (or volume) compressibility materials this matching condition cannot be satisfied as well, since the compressibility coefficient for no material is exactly equal to zero, even for diamond (0.75/TPa) and osmium (0.72/TPa), the most incompressible materials in nature.

(iii) if a ZLC axis is independently introduced into the NLC system, then minimum(α_Y,α_Z) ~ 0, and the equation (2) can always be satisfied. This means that the matching direction can be always found in the mechanical system coexisting with NLC, ZLC and PLC.

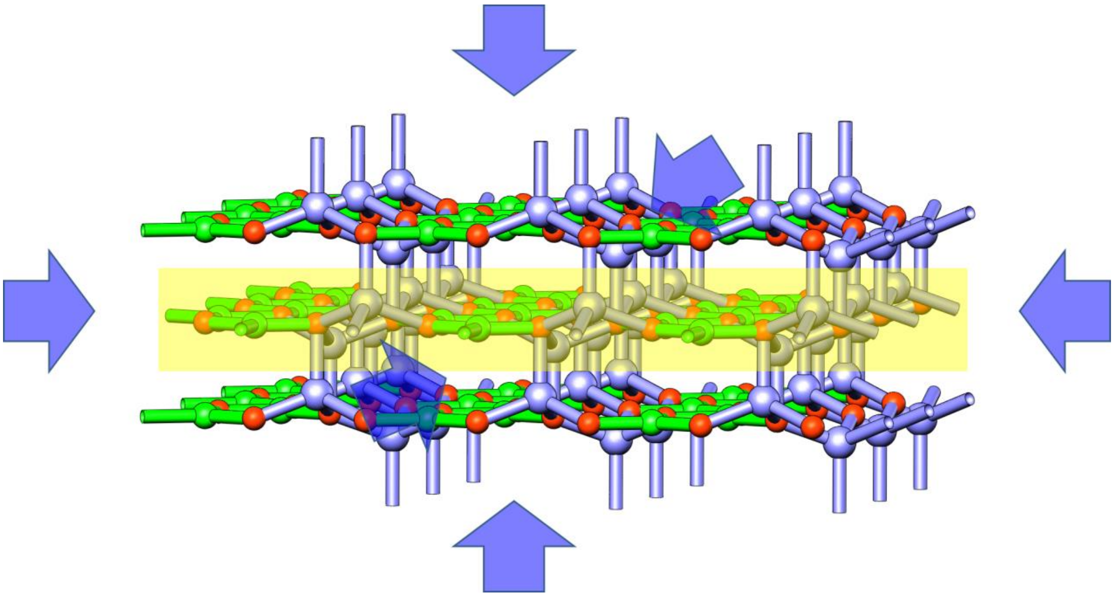

Fig. S1 ｜Structural model for finite element analysis. Lithium, boron, oxygen atoms are represented by blue, green and red balls, and Li-O and B-O bonds are represented by blue and green bars respectively. The part used to display the stress and strain is highlighted by yellow color.

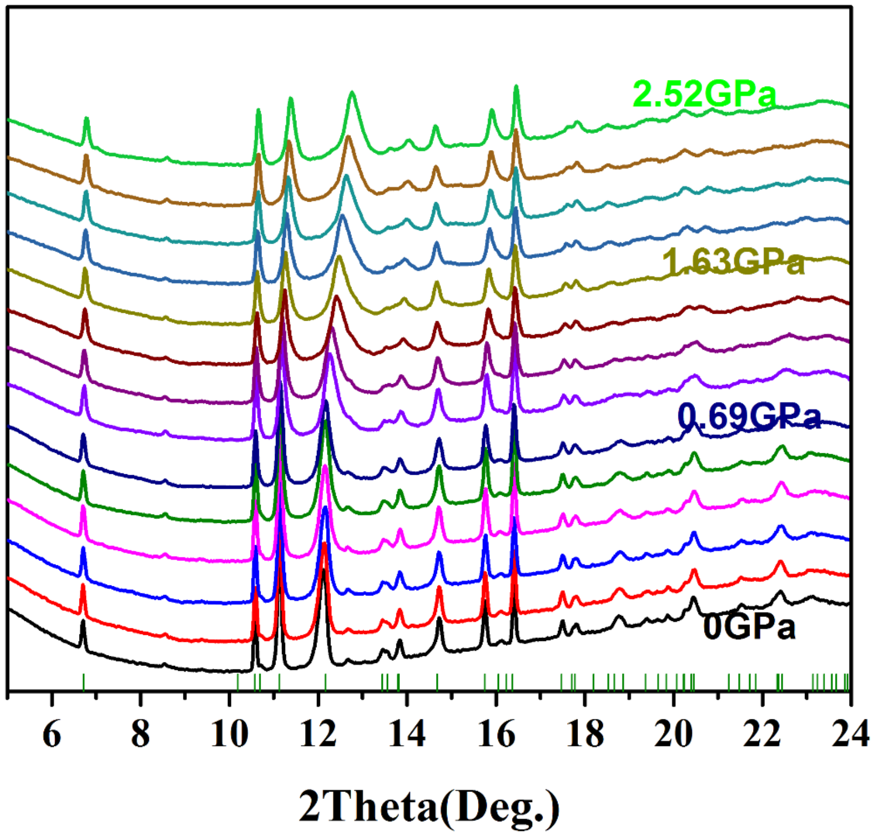

Fig. S2 | **X-ray diffraction patterns of $LiBO_2$ at various pressures.**

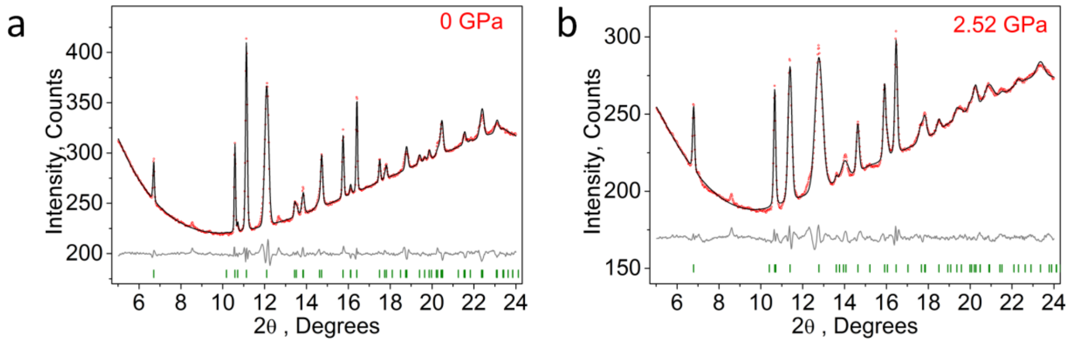

Fig. S3 | Refinement plot for the XRD patterns. a. 0GPa and **b.** 2.52GPa.

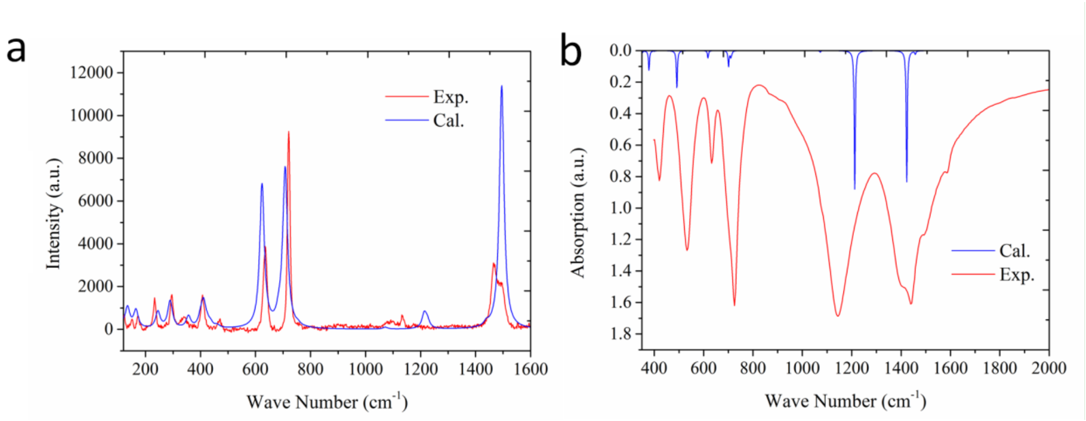

Fig. S4 | Comparison between the experimental and calculated spectra. a. Raman and **b.** infared spectra. The measured and calculated spectra are in good agreement, and almost all main peaks in the experimental spectra can be assigned in the simulated spectra, demonstrating that no defects and adsorbed species exist in the sample.

Table S1 | Refined cell parameters at respective pressures

P(GPa)	a(Å)	b(Å)	c(Å)	beta(°)	V(Å^3)
***0**	5.838(2)	4.348(1)	6.449(2)	115.12(2)	148.22
**0**	5.8275(9)	4.3454 (5)	6.4661(10)	114.5563(62)	148.931(37)
**0.12**	5.8272(13)	4.3430(9)	6.4598(14)	114.6510(54)	148.583(55)
**0.28**	5.8299(12)	4.3433(8)	6.4559(13)	114.7340(56)	148.475(51)
**0.43**	5.8292(13)	4.3426(8)	6.4504(14)	114.7955(69)	148.232(55)
**0.51**	5.8304(14)	4.3428(9)	6.4506(14)	114.8139(72)	148.252(56)
**0.69**	5.8332(18)	4.3435(11)	6.4476(19)	114.8895(85)	148.188(75)
**0.91**	5.8435(29)	4.3400(18)	6.4101(30)	115.4330(140)	146.810(120)
**1.13**	5.8486(28)	4.3395(18)	6.4026(30)	115.5750(130)	146.570(120)
**1.44**	5.8596(26)	4.3360(17)	6.3639(28)	116.1030(140)	145.200(110)
**1.63**	5.8667(26	4.3364(16)	6.3516(27)	116.3010(140)	144.860(100)
**1.98**	5.8730(32)	4.3347(24)	6.3288(37)	116.5860(130)	144.080(140)
**2.10**	5.8772(27)	4.3309(18)	6.2990(29)	116.8770(120)	143.010(110)
**2.37**	5.8770(29)	4.3291(19)	6.2839(32)	117.0060(120)	142.440(120)
**2.52**	5.8835(27)	4.3285(18)	6.2642(29)	117.2260(110)	141.850(110)

*the values determined by Zachariasen in Ref[1]

Table S2 | Calculated cell parameters at respective pressures.

P(GPa)	a(Å)	b(Å)	c(Å)	beta(°)	V(Å^3)
**0**	5.62061	4.36359	6.62551	109.077	153.573
**0.2**	5.62301	4.36146	6.60448	109.301	152.868
**0.4**	5.62423	4.36045	6.58056	109.395	152.224
**0.6**	5.63325	4.35841	6.55967	109.79	151.542
**0.8**	5.63771	4.35775	6.5394	110.016	150.954
**1**	5.65122	4.35531	6.51568	110.571	150.143
**1.2**	5.66554	4.35380	6.49318	111.155	149.371
**1.4**	5.67942	4.35239	6.47112	111.72	148.603
**1.6**	5.69000	4.35069	6.44784	112.162	147.827
**1.8**	5.70167	4.34919	6.42601	112.647	147.064
**2**	5.71187	4.34786	6.40363	113.1	146.280
**2.2**	5.72241	4.34637	6.38114	113.539	145.503
**2.4**	5.73231	4.34514	6.36057	113.969	144.765
**2.6**	5.74720	4.34329	6.33744	114.596	143.840

Table S3 | Transformation matrix between crystallographic axes *a, b, c* and mechanical principal axes *X, Y, Z*.

Experimental

$$\begin{bmatrix} X \\ Y \\ Z \end{bmatrix} = \begin{bmatrix} 0.9650 & 1 & -0.2621 \\ 0 & 1 & 0 \\ -0.5775 & 0 & -0.8264 \end{bmatrix} \begin{bmatrix} a \\ b \\ c \end{bmatrix}$$

Calculated

$$\begin{bmatrix} X \\ Y \\ Z \end{bmatrix} = \begin{bmatrix} 0.9605 & 1 & -0.2784 \\ 0 & 1 & 0 \\ -0.5795 & 0 & -0.8150 \end{bmatrix} \begin{bmatrix} a \\ b \\ c \end{bmatrix}$$

Table S4 | Theoretically refined bond lengths and angles at various pressures.

Pressure	Average B-O bond	O-B-O	Li-O bond outside $[LiBO_2]_\infty$ plane	Average Li-O bond inside $[LiBO_2]_\infty$ plane	∠O-Li-O
**0**	1.3712	133.202	1.9947	1.9578	127.435
**0.12**	1.3709	133.199	1.9930	1.9567	127.504
**0.28**	1.3709	133.187	1.9930	1.9566	127.667
**0.43**	1.3709	133.16	1.9923	1.9549	127.815
**0.51**	1.3709	133.138	1.9922	1.9552	127.802
**0.69**	1.3709	133.193	1.9920	1.9558	127.895
**0.91**	1.3704	133.120	1.9876	1.9517	128.821
**1.13**	1.3705	133.076	1.9884	1.9508	129.127
**1.44**	1.3700	133.021	1.9849	1.9471	130.121
**1.63**	1.3701	133.025	1.9840	1.9470	130.500
**1.98**	1.3700	132.979	1.9818	1.9449	131.057
**2.10**	1.3695	132.909	1.9774	1.9416	131.621
**2.37**	1.3693	132.875	1.9754	1.9393	131.842
**2.52**	1.3692	132.868	1.9746	1.9381	132.366

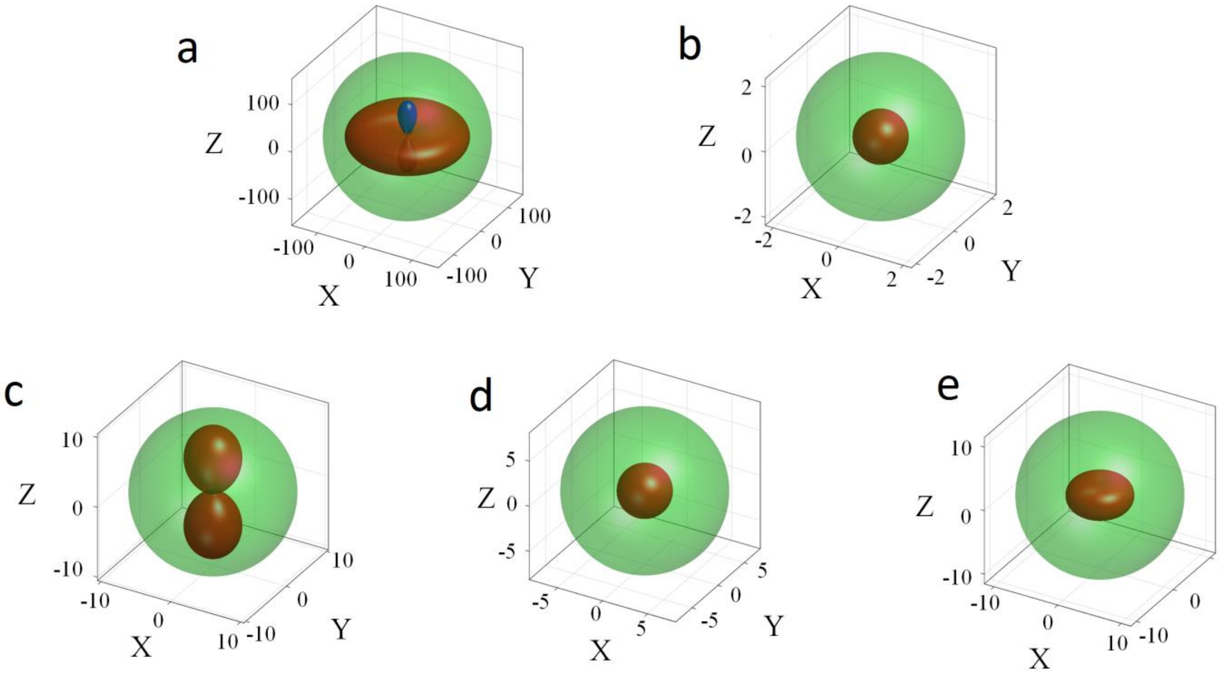

Fig. S5 | **Matching curves between linear and volume compressibilities** in **a.** $Ag_3Co(CN)_6$ **, b.** diamond**, c.** graphite**, d.** copper and **e.** quartz. The compressibility surfaces are plotted based on the data listed in Table S5. Positive and negative linear compressibilities are represented by red and blue surfaces, and volume compressibility is represented by green sphere, respectively. Clearly, for all materials no intersecting line exists between the volume and linear compressibility surfaces, indicating that they cannot squeeze the three-dimensional volume compressibility into one dimension.

Table S5 | Linear compressibilities (α_l) and volume compressibility (α_V) in $LiBO_2$, $Ag_3Co(CN)_6$, diamond, graphite, copper, and quartz, as well as the relative fluctuation of flux density and optimal transmission direction in these materials as they move from sea level to the Mariana Trench. The minimum relative fluctuation of flux density is defined as the product of the compressibility of transmission cross-section and the pressure at Mariana Trench (0.11GPa) along the optimal direction among the integer angles (in degree) closest to the exact matching direction between volume and linear compressibilities. All the linear compressibility values at the integer angles closest to the matching curve in $LiBO_2$ are listed in Table S6. For other materials the optimal transmission direction is along the largest PLC axis, since its compressibility value has the smallest difference with the volume compressibility.

	**α_l (X) (/TPa)**	**α_l (Y) (/TPa)**	**α_l (Z) (/TPa)**	**α_V (/TPa)**	Minimum relative fluctuation of flux density	Optimal transmission direction for the minimum relative fluctuation of flux density **(θ,φ)**	References for compressibilities
$LiBO_2$	-5.33	1.66	25.63	21.96	$\leq 1.10\times10^{-6}$	(20°, 30°), (21°, 73°)	This work
$Ag_3Co(CN)_6$	115	115	-75	155	4.40×10^{-2}	(90°, φ)	Ref. 2
diamond	0.75	0.75	0.75	2.25	1.65×10^{-4}	arbitrary (θ, φ)	Ref.3
graphite	0.70	0.70	9.14	10.54	1.54×10^{-4}	(0°, φ)	Ref.4
copper	2.71	2.71	2.71	8.13	5.96×10^{-4}	arbitrary (θ, φ)	Ref.5
quartz	4.7	4.7	2.11	11.51	7.49×10^{-4}	(90°, φ)	Ref.6

Table S6 | Linear compressibility values at the integer angles (in degree) closest to near the matching curve between volume and linear compressibilities in $LiBO_2$. Because the matching curve is continuous in space, for each integer polar angle φ there has a corresponding integer azimuth angle θ closest to the curve. Since the matching curve is symmetry with respect to (*X*, *Y*), (*X*, *Z*) and (*Y*, *Z*) planes, only the values in the first quadrant (i.e., 0≤θ≤90°, and 0≤φ≤90°) are displayed, and those in the other quadrants can be easily obtained by symmetry operation. Clearly, the optimal linear compressibility are located at (22°, 30°) and (21°, 73°) with 21.962/TPa and 21.970/TPa, respectively (highlighted by blue color). Both values just deviate from the volume compressibility (21.96/TPa) by no more than 0.01/TPa.

**(θ,φ) (°,°)**	linear compressibility **(/TPa)**	**(θ,φ) (°,°)**	linear compressibility (/TPa)	**(θ,φ) (°,°)**	linear compressibility (/TPa)
(20, 0)	21.905	(22, 31)	21.944	(22, 61)	22.006
(20, 1)	21.906	(22, 32)	22.025	(22, 62)	21.976
(20, 2)	21.909	(22, 33)	22.054	(22, 63)	21.945
(20, 3)	21.915	(22, 34)	22.081	(22, 64)	21.914
(20, 4)	21.922	(23, 35)	22.107	(22, 65)	21.882
(20, 5)	21.931	(23, 36)	21.823	(22, 66)	21.850
(20, 6)	21.943	(23, 37)	21.847	(22, 67)	21.817
(20, 7)	21.956	(23, 38)	21.869	(21, 68)	22.115
(20,8)	21.972	(23, 39)	21.888	(21, 69)	22.085
(20, 9)	21.989	(23, 40)	21.905	(21, 70)	22.056
(20 10)	22.008	(23, 41)	21.919	(21, 71)	22.026
(20, 11)	22.028	(23, 42)	21.931	(21, 72)	21.998
(20,12)	22.051	(23, 43)	21.940	**(21, 73)**	**21.970**
(20, 13)	22.074	(23, 44)	21.946	(21, 74)	21.943
(20, 14)	22.099	(23, 45)	21.950	(21, 75)	21.916
(21, 15)	21.784	(23, 46)	21.951	(21, 76)	21.891
(21, 16)	21.814	(23, 47)	21.950	(21, 77)	21.867
(21, 17)	21.845	(23, 48)	21.946	(21, 78)	21.844
(21, 18)	21.877	(23, 49)	21.939	(21, 79)	21.823
(21, 19)	21.909	(23, 50)	21.93	(21, 80)	21.804
(21, 20)	21.942	(23, 51)	21.918	(21, 81)	22.134
(21, 21)	21.976	(23, 52)	21.904	(21, 82)	22.119
(21, 22)	22.010	(23, 53)	21.887	(21, 83)	22.106
(21, 23)	22.044	(23, 54)	21.868	(21, 84)	22.095
(21, 24)	22.078	(23, 55)	21.847	(21, 85)	22.086
(21, 25)	22.112	(23, 56)	21.824	(21, 86)	22.078
(22, 26)	21.823	(22, 57)	22.112	(21, 87)	22.072
(22, 27)	21.859	(22, 58)	22.088	(21, 88)	22.068
(22, 28)	21.894	(22, 59)	22.062	(21, 89)	22.066
(22, 29)	21.929	(22, 60)	22.034	(21, 90)	22.066
**(22, 30)**	**21.962**				

Table S7 | Calculated UV absorption edge (λ_{cutoff}) and band gap (E_g) in $LiBO_2$ at various pressures.

**Pressure (GPa)**	λ_{cutoff} (nm)	Eg (eV)
**0**	174.648	7.10
**0.2**	173.670	7.14
**0.4**	173.427	7.15
**0.6**	172.943	7.17
**0.8**	172.462	7.19
**1**	171.745	7.22
**1.2**	171.034	7.25
**1.4**	170.564	7.27
**1.6**	169.863	7.30
**1.8**	169.168	7.33
**2**	168.707	7.35
**2.2**	168.022	7.38
**2.4**	167.116	7.42
**2.6**	165.997	7.47

Reference

1. Zachariasen, W. H. Crystal structure of lithium metaborate. *Acta Crystallogr.* **17**, 749-751 (1964).

2. Goodwin, A. L., Keen, D. A. & Tucker, M. G. Large negative linear compressibility of Ag_3 $[Co(CN)]_6$. *Proc. Natl. Acad. Sci. USA* **105**, 18708-18713(2008).

3. Occelli, F., Loubeyre, P. & Letoullec, R. Properties of diamond under hydrostatic pressures up to 140 GPa. *Nat. Mater.* **2**, 151-154(2003).

4. Yagi, T., Utsumi, W., Yamakata, M., Kikegawa, T. & Shimomura, O. High-pressure *in situ* X-ray-diffraction study of the phase-transformation from graphite to hexagonal diamond at room-temperature. *Phys. Rev. B* **46**, 6031-6039 (1992).

5. Engineering ToolBox: Tools and Basic Information for Design, Engineering and Construction of Technical Applications (https://www.engineeringtoolbox.com/bulk-modulus-metals-d_1351.html).

6. Hazen, R. M., Finger, L. W., Hemley, R. J. & Mao, H. K. High-pressure crystal-chemistry and amorphization of alpha-quartz. *Solid State Commun.* 1989, **72**, 507-511(1989).
