# Supplementary material for: Anomalous mechanical materials squeezing three-dimensional volume compressibility into one dimension
Source: Nat Commun. 2020 Nov 5;11:5593. doi: 10.1038/s41467-020-19219-5 (PMC7644688; doi:10.1038/s41467-020-19219-5)
Supplement: Supplementary file 2 — Description of Additional Supplementary Files [file 41467_2020_19219_MOESM2_ESM.docx]

Description of Additional Supplementary Information

Title: Supplementary Data 1

Description: The refined crystal structure files (CIF) and checkcif report for the crystal structures at respective pressure.
