# Supplementary material for: Anomalous mechanical materials squeezing three-dimensional volume compressibility into one dimension
Source: Nat Commun. 2020 Nov 5;11:5593. doi: 10.1038/s41467-020-19219-5 (PMC7644688; doi:10.1038/s41467-020-19219-5)

# checkCIF/PLATON report

You have not supplied any structure factors. As a result the full set of tests cannot be run.

THIS REPORT IS FOR GUIDANCE ONLY. IF USED AS PART OF A REVIEW PROCEDURE FOR PUBLICATION, IT SHOULD NOT REPLACE THE EXPERTISE OF AN EXPERIENCED CRYSTALLOGRAPHIC REFEREE.

No syntax errors found. CIF dictionary Interpreting this report

## Datablock: a

---

```
Bond precision:  O- B = 0.0200 A         Wavelength=0.61992

Cell:            a=5.8332(18)    b=4.3435(11)        c=6.4476(19)
                 alpha=90        beta=114.890(8)     gamma=90
Temperature:     300 K

                Calculated                 Reported
Volume          148.19(7)                  148.19(8)
Space group     P 21/c                     P21/c
Hall group      -P 2ybc                    -P 2ybc
Moiety formula  B2 O4, 2(Li)               B2 O4, 2(Li)
Sum formula     B2 Li2 O4                  B2 Li2 O4
Mr              99.50                      99.50
Dx,g cm-3       2.230                      2.226
Z               2                          2
Mu (mm-1)       0.144                      1.765
F000            96.0                       0.0
F000'           96.05
h,k,lmax        3,2,4
Nref            46
Tmin,Tmax
Tmin'

Correction method= Not given

Data completeness= 0.000          Theta(max)=

R(reflections)=                   wR2(reflections)=

S =                         Npar=
```

---

The following ALERTS were generated. Each ALERT has the format
**test-name_ALERT_alert-type_alert-level**.
Click on the hyperlinks for more details of the test.

---

### Alert level C

```
CRYSR01_ALERT_1_C  _exptl_crystal_size_rad not in the CIF when expected.
```

---

### Alert level G

```
ABSMU01_ALERT_1_G  Calculation of _exptl_absorpt_correction_mu
                not performed for this radiation type.
PLAT004_ALERT_5_G Polymeric Structure Found with Maximum Dimension          1 Info
PLAT092_ALERT_4_G Check: Wavelength Given is not Cu,Ga,Mo,Ag,In Ka    0.61992 Ang.
```

---

```
   0 ALERT level A = Most likely a serious problem - resolve or explain
   0 ALERT level B = A potentially serious problem, consider carefully
   1 ALERT level C = Check. Ensure it is not caused by an omission or oversight
   3 ALERT level G = General information/check it is not something unexpected

   2 ALERT type 1 CIF construction/syntax error, inconsistent or missing data
   0 ALERT type 2 Indicator that the structure model may be wrong or deficient
   0 ALERT type 3 Indicator that the structure quality may be low
   1 ALERT type 4 Improvement, methodology, query or suggestion
   1 ALERT type 5 Informative message, check
```

---

It is advisable to attempt to resolve as many as possible of the alerts in all categories. Often the minor alerts point to easily fixed oversights, errors and omissions in your CIF or refinement strategy, so attention to these fine details can be worthwhile. In order to resolve some of the more serious problems it may be necessary to carry out additional measurements or structure refinements. However, the purpose of your study may justify the reported deviations and the more serious of these should normally be commented upon in the discussion or experimental section of a paper or in the "special_details" fields of the CIF. checkCIF was carefully designed to identify outliers and unusual parameters, but every test has its limitations and alerts that are not important in a particular case may appear. Conversely, the absence of alerts does not guarantee there are no aspects of the results needing attention. It is up to the individual to critically assess their own results and, if necessary, seek expert advice.

**Publication of your CIF in IUCr journals**

A basic structural check has been run on your CIF. These basic checks will be run on all CIFs submitted for publication in IUCr journals (*Acta Crystallographica*, *Journal of Applied Crystallography*, *Journal of Synchrotron Radiation*); however, if you intend to submit to *Acta Crystallographica Section C* or *E* or *IUCrData*, you should make sure that full publication checks are run on the final version of your CIF prior to submission.

**Publication of your CIF in other journals**

Please refer to the *Notes for Authors* of the relevant journal for any special instructions relating to CIF submission.

---

**PLATON version of 07/08/2019; check.def file version of 30/07/2019**

**Datablock a** - ellipsoid plot

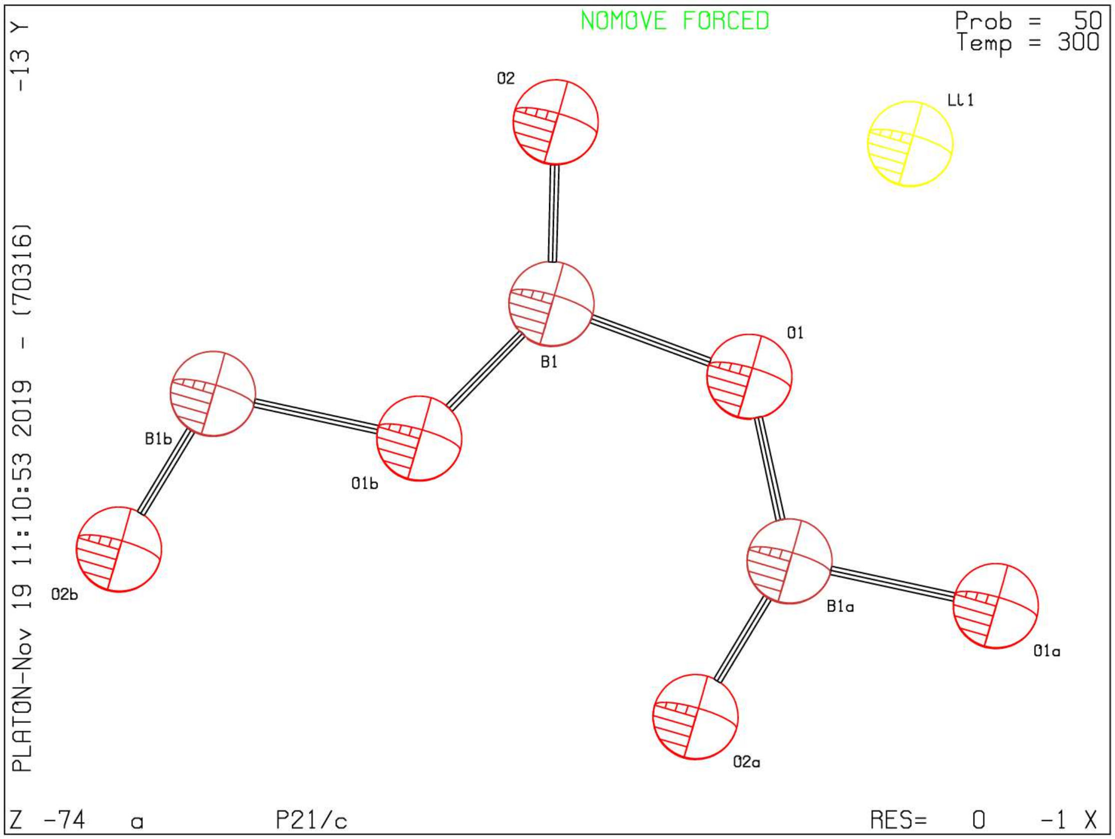

Supplement: Supplementary file 3 — Supplementary Data 1 [file 41467_2020_19219_MOESM3_ESM.zip › CIF-files/checkcif_LiBO2_0p69GPa.pdf]
